# Reservoir quality evaluation of tight carbonate gas reservoirs using fractal–multifractal pore-structure metrics: a case study from the Taiyuan Formation, Ordos Basin, China

**DOI:** 10.1038/s41598-026-64360-8

**Published:** 2026-09-07

**Authors:** Yang Wang, Wenchao Pei, Li Jia, Long Guo, Jie Lei, Yunlong Zhang, Shuyun Xie

**Affiliations:** 1 State Key Laboratory of Geological Processes and Mineral Resources, School of Earth and Planetary Sciences, China University of Geosciences, Wuhan, 430074 China; 2 Research Institute of Exploration and Development, Changqing Oilfeld Company, PetroChina, Xi’an, 710018 Shaanxi China; 3 School of Mathematics and Physics, China University of Geosciences, Wuhan, 430074 China

**Keywords:** Tight carbonate gas reservoir, Reservoir-quality evaluation, Mercury intrusion porosimetry, Fractal-multifractal analysis, Pore-throat heterogeneity, Taiyuan Formation, Ordos Basin, Energy science and technology, Engineering, Solid Earth sciences

## Abstract

Tight carbonate gas reservoirs commonly contain appreciable storage space but highly variable flow capacity, making reservoir-quality evaluation and favorable-interval screening challenging in exploration and development. This study investigates 10 representative limestone core samples from the Taiyuan Formation, Ordos Basin, China, using high-pressure mercury intrusion data integrated with segmented single-fractal and multifractal analyses to identify pore-structure metrics relevant to reservoir evaluation. The results show that the pore systems exhibit segmented fractal characteristics: the low-pressure fractal dimension  ${D}_{1}$  is more sensitive to the development of fracture-related macropore pathways and may therefore be associated with seepage potential, whereas the high-pressure fractal dimension  ${D}_{2}$  mainly reflects the roughness and complexity of matrix-controlled storage pores. Furthermore, multifractal parameters provide a quantitative description of the overall heterogeneity of the pore system. The spectral width ($\Delta \alpha$) reflects the intensity of heterogeneity, while the asymmetry index (*R*) helps distinguish whether the pore structure is more strongly influenced by large seepage pores or small storage pores. When combined with pore-throat attributes and multivariate classification, these metrics divide the reservoir into three resource-quality classes and suggest that intervals with high ${D}_{1}$, positive *R*, and moderate $\Delta \alpha$ are more favorable pore-structure conditions for fluid mobility. The proposed workflow therefore provides a preliminary pore-structure-based framework for reservoir-quality evaluation and favorable-interval screening in tight carbonate gas reservoirs.

## Introduction

The exploration and development of tight carbonate reservoirs have increasingly emerged as a strategic focal point in global unconventional oil and gas research^1–3^. However, the efficient exploitation of these low-permeability reservoirs is frequently constrained by the strong heterogeneity and intricate complexity of microscopic pore structures, which act as the primary geological factors limiting productivity^4–7^. In the Ordos Basin, the Permian Taiyuan Formation limestone represents a strategic successor for natural gas exploration, however, its resource potential is severely challenged by intricate pore-throat networks^8,9^. Influenced by complex sedimentation, diagenesis, and tectonic evolution, these reservoirs develop multi-scale irregularities characterized by the coexistence of dissolution pores and micro-fractures which fundamentally control the fluid storage capacity and flow capacity within the porous media^10–12^. Consequently, the quantitative characterization of pore structure heterogeneity is essential for clarifying the mechanisms governing macroscopic physical properties and for the accurate evaluation of reservoir quality^13^.

Mercury intrusion porosimetry (MIP) is commonly utilized to determine the pore structure distributions of rock formations^14–18^. Early interpretations primarily relied on basic characteristic parameters, such as displacement pressure (${P}_{d}$) and median pore-throat radius (${r}_{50}$), to estimate reservoir storage capacity^18–22^. To establish a more robust link between static pore geometry and dynamic fluid flow, the focus subsequently shifted towards identifying the effective pore throat radius, notably through the ${R}_{apex}$ method based on Swanson’s parameter^23,24^. While the ${R}_{apex}$ method successfully correlates the apex of the mercury saturation-pressure hyperbola with permeability in conventional reservoirs^25–27^, these Euclidean-geometry-based parameters often fall short in describing the strong heterogeneity inherent in tight or complex formations^18,28–30^. Consequently, to address the multiscale irregularity of pore systems, fractal theory has been increasingly adopted as a powerful characterization tool^18,28–33^.

Numerous studies regarding pore structure heterogeneity have been completed based on various fractal theories using experimental data obtained from experiments such as, among others, high pressure mercury injection^26,34,35^, nitrogen or carbon dioxide gas adsorption^36–39^, Nuclear Magnetic Resonance (NMR)^37,40,41^, and digital rock physics such as CT or FIB data^42–46^. In the analysis of capillary pressure data, fractal geometry^47^ has primarily been employed to calculate fractal dimensions and establish quantitative relationships between these dimensions and reservoir physical properties^48,49^. Various fractal models based on single fractal theory have been developed to extract fractal dimensions from mercury injection data^50–52^. However, single fractal theory fundamentally describes the global integrity characteristics of pore structures^48^. While suitable for application to homogeneous reservoirs, it generally fails to define local pore structure variations or reflect other comprehensive and detailed information^43^. Furthermore, the “segmented fractal” phenomenon—where data exhibits different fractal dimensions across different pressure ranges—frequently occurs when applying fractal theory to mercury injection data^53–55^. This phenomenon demonstrates the inherent difficulty of characterizing the complexity of the entire pore system using a single fractal dimension.

To address the limitations of single fractal theory in describing local pore size distribution characteristics, multifractal analysis has been conducted to quantify the complexity of pore size distributions. Multifractal theory partitions objects across different scales to obtain distribution characteristics^56,57^. Consequently, it is likely to characterize the complex and heterogeneous behavior of a reservoir more effectively than single fractal theory^48^. Multifractal theory has been widely applied to investigate the effect of pore size changes on reservoir physical properties^58^. For instance, multifractal analysis of soil pore structure has been extensively studied to characterize soil structural stability and soil surface evolution stages^59^. It has also been confirmed as a useful tool for characterizing the internal complexity of coal and amplifying the differences in pore size distributions between different coals^13^. Additionally, quantitative characterization of irregular microscopic pore structures in different rock types has been performed on the basis of 2D images^40,60,61^.

Recent studies have further expanded fractal and multifractal approaches toward full-scale pore-size characterization, integrated pore-architecture analysis, and adsorption-related pore heterogeneity. Liu et al. developed a fractal strategy for improved characterization of full-scale pore-size distribution, highlighting the importance of cross-scale pore description^62^. Cao et al. proposed an integrated multifractal framework to characterize full-scale pore architecture and reservoir-controlling mechanisms in deep low-rank coal^63^. In addition, recent studies have extended fractal and multifractal approaches to methane adsorption in coal pores and fractal Langmuir adsorption modeling, showing the potential of these methods for linking pore heterogeneity with gas occurrence and adsorption behavior^64,65^. However, most of these recent advances focus on coal or adsorption-dominated pore systems, whereas tight carbonate gas reservoirs with coupled pore–fracture systems remain less systematically investigated using an integrated segmented-fractal and multifractal workflow.

In this study, we apply both single fractal and multifractal theories to investigate the variability and heterogeneity of pore structures in the Taiyuan Formation limestones based on mercury intrusion curves. The specific objectives are to calculate fractal parameters to differentiate seepage and storage capabilities, and to employ Principal Component Analysis (PCA) for preliminary reservoir classification. By integrating segmented single-fractal parameters, multifractal spectrum descriptors, mercury-intrusion pore-throat attributes, and exploratory multivariate classification, this work aims to establish a quantitative framework combining the asymmetry index (*R*) with fractal dimensions (${D}_{1}$) as key indicators for identifying exploration ‘sweet spots’ in tight carbonate gas reservoirs of the Taiyuan Formation.

It should be emphasized that the purpose of this study is not to propose a new mathematical fractal or multifractal model, but to provide an application-oriented integration of segmented single-fractal indices, multifractal spectrum descriptors, MIP-derived pore-throat attributes, and exploratory multivariate classification for reservoir-quality evaluation of tight carbonate gas reservoirs. This integrated workflow is used to distinguish seepage-related macropore/fracture features from storage-related matrix-pore heterogeneity within the studied Taiyuan Formation limestones.

## Geological setting

The Ordos Basin, situated on the western margin of the North China Plate, is characterized as a nearly rectangular, multi-cycle, poly-history composite superimposed basin^66,67^. The current study area is located in the east-central portion of the basin, specifically on the eastern flank of the Yishan Slope structural unit. Influenced by the Caledonian tectonic cycle, the North China Plate underwent structural uplift during the Middle to Late Ordovician, which led to the termination of the epicontinental marine sedimentary system within the basin and its surrounding regions. By the Late Carboniferous, the environment had transitioned into a transitional marine-terrestrial sedimentary setting. During the Early Permian, the subduction and consumption of oceanic crust toward the North China Plate from both the northern and southern margins controlled the formation of a structural framework characterized by dual-margin uplifts and a central depression. Concurrently, sediment supply from both margins converged toward the central shallow sea, establishing a transitional marine-terrestrial delta-tidal flat sedimentary system composed of northern sandstones, southern sandstones, and central limestones.

Based on the lithological assemblages of limestone, sandstone, mudstone, and coal, the Taiyuan Formation is divided from bottom to top into the Miaogou, Mao’ergou, Xiedao, and Dongdagao members^68^. The Miaogou Member primarily consists of mudstone and sandstone, with thin layers of carbonaceous mudstone observed at the top. The Maoergou and Xiehao Members represent the primary intervals for limestone development within the study area. Thick-bedded limestones generally develop in the lower to middle portions of these members, transitioning rapidly upward into mudstone and coal. The Dongdagao Member features 1–2 m thick argillaceous limestone in its lower section, which gradually grades upward into carbonaceous mudstone. This member is locally developed across the study area.

**Fig. 1:**
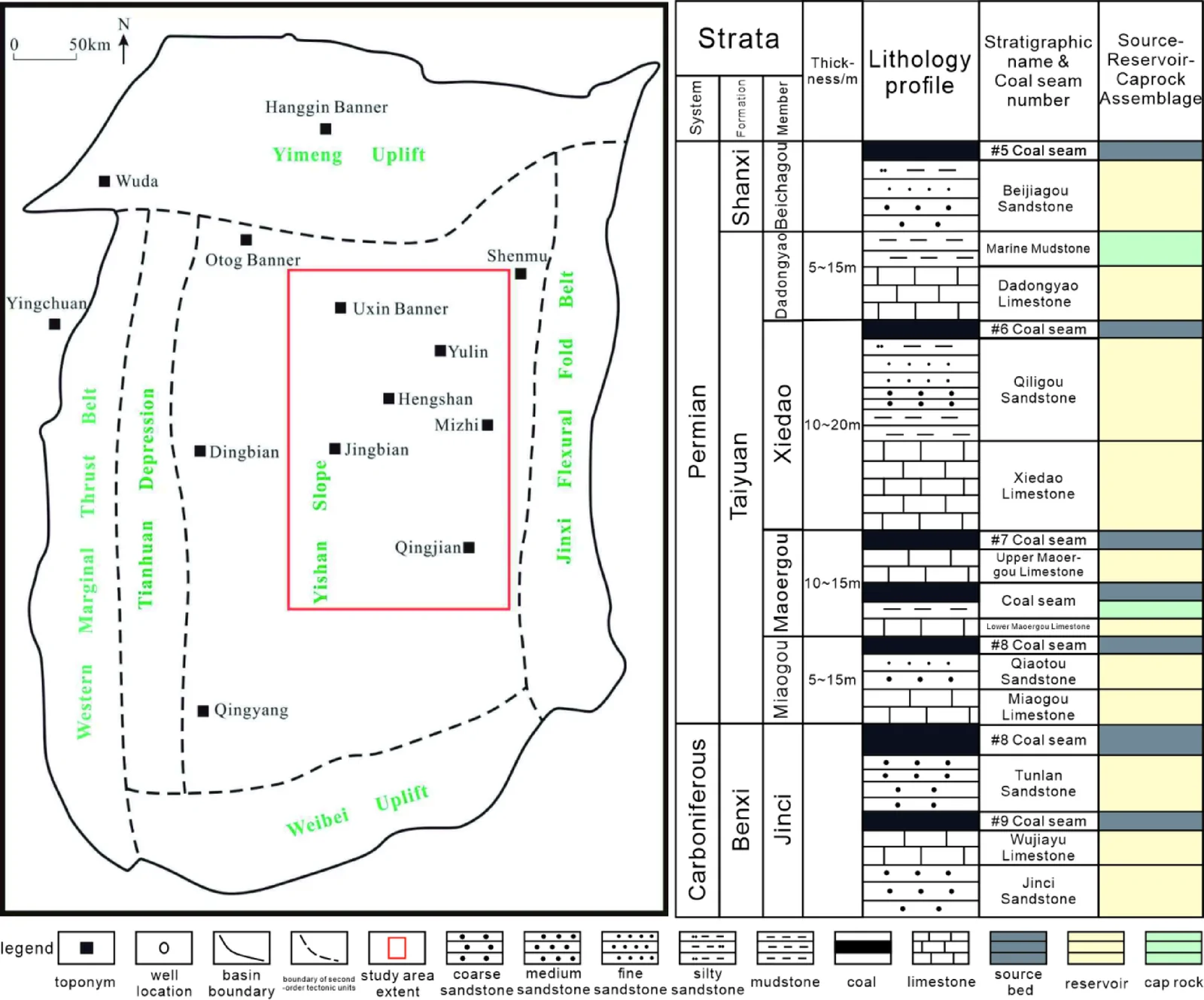
Tectonic geological conditions of the Ordos Basin and comprehensive stratigraphic column of the Taiyuan Formation^69^.

The study area is located in the eastern margin of the Ordos Basin, within the Yishan Slope structural unit (Fig. 1). The Taiyuan Formation limestone in this region represents an important target for tight gas exploration. Core samples analyzed in this study were obtained from exploration wells drilled in this area. The samples cover different lithofacies and pore structure types, providing a representative dataset for investigating pore structure heterogeneity. Thin-section observations and mercury-intrusion results indicate that the samples include the main reservoir space and pore-throat types of the Taiyuan Formation limestones. All samples were collected from depths corresponding to the Taiyuan Formation and were selected to ensure variability in porosity and permeability.

## Methods

### Single fractal theory

Early fractal models based on MIP data, such as the thermodynamic model^50^ and the geometric model^51^, have successfully quantified the irregularity of pore spaces. However, these traditional models typically assume the porous medium consists of parallel capillary bundles, focusing solely on the fractal characteristics of the pore volume (${D}_{f}$) while ignoring the complexity of fluid flow paths^70^.

In tight reservoirs, the tortuosity of flow channels significantly impacts permeability. To address this, the concept of fractal tortuosity dimension (${D}_{T}$) was introduced to quantify the convoluted nature of streamlines in porous media^71^. By integrating both the pore space fractal dimension (${D}_{f}$) and the tortuosity fractal dimension (${D}_{T}$), Li (2010) and Su et al. (2018) derived a generalized fractal model for mercury intrusion^52,72^:

1 $$\begin{array}{*{20}c} {\log S_{Hg} \propto \left( {D_{f} + D_{T} - 3} \right)\log \left( {P_{c} } \right)} \\ \end{array}$$

where ${S}_{Hg}$ is the mercury saturation and ${P}_{c}$ is the capillary pressure. The slope *K* of the $\mathrm{l}\mathrm{o}\mathrm{g}({S}_{Hg})$ versus $\mathrm{l}\mathrm{o}\mathrm{g}({P}_{c})$ plot is linearly related to the fractal dimensions. Empirical studies on tight carbonate reservoirs reveal that the $\mathrm{l}\mathrm{o}\mathrm{g}({S}_{Hg})$–$\mathrm{l}\mathrm{o}\mathrm{g}({P}_{c})$ curves often exhibit a distinct “bilinear” or segmented characteristic rather than a single straight line^73^. This segmentation indicates the existence of two distinct pore systems controlled by different geological mechanisms: Low-pressure segment (${D}_{1}$): Corresponds to large pores and micro-fractures. A higher ${D}_{1}$ value in this region typically reflects the structural complexity of macroscopic seepage channels, which dominate the fluid flow capacity^73,74^. High-pressure segment (${D}_{2}$): Corresponds to mesopores and matrix pores. The fractal dimension ${D}_{2}$ characterizes the roughness and irregularity of these storage spaces, which govern the reservoir’s storage capacity^70^. This segmented fractal approach allows for a more detailed decoupling of the reservoir’s seepage and storage capabilities compared to single-slope models.

### Multifractal theory

Multifractal theory describes the local characteristics of fractal structures through the singularity exponent, investigating global properties from a local perspective^75^. This multifractal model analyzes the pore distribution characteristics of rock samples based on mercury intrusion porosimetry to determine their oil and gas conductivity. A prerequisite for constructing this one-dimensional multifractal model is defining the measurement interval and dividing it into equidistant segments^76^.

In this model, the pore throat diameter *D* is first logarithmically transformed to obtain the parameter $\mathrm{l}\mathrm{o}\mathrm{g}D$, defining the measurement interval $J=\left[\mathrm{l}\mathrm{o}\mathrm{g}{D}_{min},\mathrm{l}\mathrm{o}\mathrm{g}{D}_{max}\right]$. The measurement interval *J* is divided into $N={2}^{k}$ equal sub-intervals of size $\varepsilon =J\times {2}^{-k}$, where ${J}_{i}$ represents the *i*-th sub-interval of the measurement interval *J*. As the value of *k* increases, the size of the divided sub-intervals gradually decreases.

Defining ${v}_{i}\left(\varepsilon \right)$ as the pore volume within sub-interval ${J}_{i}$, ${v}_{i}\left(\varepsilon \right)$ can be derived from the pore volume distribution function $dv/d\mathrm{l}\mathrm{o}\mathrm{g}D$ obtained via MIP:

2 $$\begin{array}{*{20}c} {v_{i} \left( \varepsilon \right) = \mathop \smallint \limits_{{\log D_{i} }}^{{\log D_{i + 1} }} \frac{dv}{{d\log D}}d\log D} \\ \end{array}$$

To obtain sufficient data for ${v}_{i}\left(\varepsilon \right)$ to conduct multifractal analysis, this study applies cubic spline interpolation to the pore size distribution function $dv/d\mathrm{l}\mathrm{o}\mathrm{g}D$. Based on the pore volume parameter ${v}_{i}\left(\varepsilon \right)$, the probability mass ${P}_{i}\left(\varepsilon \right)$ of the pore volume within sub-interval ${J}_{i}$ relative to the total pore volume is defined as:

Cubic spline interpolation was used only to reconstruct a continuous pore-size distribution from discrete MIP data for calculating the probability measure and partition function. Because the same interpolation procedure was applied consistently to all samples, the resulting multifractal parameters are interpreted as comparative statistical descriptors within the present dataset rather than universal constants.

3 $$\begin{array}{*{20}c} {P_{i} \left( \varepsilon \right) = \frac{{v_{i} \left( \varepsilon \right)}}{{\mathop \sum \nolimits_{i = 1}^{N} v_{i} \left( \varepsilon \right)}}} \\ \end{array}$$

Among various multifractal calculation methods, the method of moments is widely recommended due to its robustness in avoiding numerical instabilities often associated with the box-counting method^77^. Therefore, this study adopts the method of moments, defining the partition function $\chi \left(q,\varepsilon \right)$ using the pore volume probability in sub-interval ${J}_{i}$:

4 $$\begin{array}{*{20}c} {\chi \left( {q,\varepsilon } \right) = \mathop \sum \limits_{i = 1}^{N} P_{i} \left( \varepsilon \right)^{q} } \\ \end{array}$$

In the equation, *q* represents the order of the moment. When *q>0*, $\chi \left(q,\varepsilon \right)$ reflects the characteristics of regions with large ${P}_{i}\left(\varepsilon \right)$, highlighting the features of large pore spaces; when *q<0*, $\chi \left(q,\varepsilon \right)$ reflects the characteristics of regions with small ${P}_{i}\left(\varepsilon \right)$, highlighting the spatial features of small pores and micropores. Simultaneously, $\chi \left(q,\varepsilon \right)$ and *ɛ* follow a power-law distribution relationship:

5 $$\begin{array}{*{20}c} {\chi \left( {q,\varepsilon } \right) \propto \varepsilon^{\tau \left( q \right)} } \\ \end{array}$$

where $\tau \left( q \right)$ is the mass exponent function, which can be expressed as:

6 $$\begin{array}{*{20}c} {\tau \left( q \right) = \mathop {\lim }\limits_{\varepsilon \to 0} \frac{{{\mathrm{lg}}\left( {\mathop \sum \nolimits_{i = 1}^{N} P_{i} \left( \varepsilon \right)^{q} } \right)}}{\log \left( \varepsilon \right)} = \left( {q - 1} \right)D\left( q \right)} \\ \end{array}$$

where $D_{q}$ is the generalized dimension, which can be expressed mathematically as:

7 $$\begin{array}{*{20}c} {D\left( q \right) = \mathop {\lim }\limits_{\varepsilon \to 0} \frac{1}{q - 1}\frac{{{\mathrm{lg}}\left( {\mathop \sum \nolimits_{i = 1}^{N} P_{i} \left( \varepsilon \right)^{q} } \right)}}{\log \left( \varepsilon \right)}} \\ \end{array}$$

When *q = 1*, $D_{1}$ is defined as:

8 $$\begin{array}{*{20}c} {D\left( 1 \right) = \mathop {\lim }\limits_{\varepsilon \to 0} \frac{{\mathop \sum \nolimits_{i = 1}^{N} P_{i} \left( \varepsilon \right)\log \left( {P_{i} \left( \varepsilon \right)} \right)}}{\log \left( \varepsilon \right)}} \\ \end{array}$$

When $\varepsilon \to 0$, $P_{i} \left( \varepsilon \right)$ is approximately uniformly distributed within each sub-interval, and a power-law relationship also exists between $P_{i} \left( \varepsilon \right)$ and *ɛ*:

9 $$\begin{array}{*{20}c} {P_{i} \left( \varepsilon \right) \propto \varepsilon^{\alpha } } \\ \end{array}$$

where *α* is the singularity strength (or index). Defining $N_{\alpha } \left( \varepsilon \right)$ as the number of sub-intervals with singularity strength between *α* and $\alpha + d\alpha$, the following power-law relationship exists between $N_{\alpha } \left( \varepsilon \right)$ and *ɛ*:

10 $$\begin{array}{*{20}c} {N_{\alpha } \left( \varepsilon \right) \propto \varepsilon^{ - f\left( \alpha \right)} } \\ \end{array}$$

where $f\left(\alpha \right)$ is the multifractal spectrum function of the singularity index *α*. *α* and $f\left(\alpha \right)$ can be obtained via Legendre transformation^42,60^:

11 $$\begin{array}{*{20}c} {\alpha \left( q \right) = \frac{\partial \tau \left( q \right)}{{\partial q}}} \\ \end{array}$$

12 $$\begin{array}{*{20}c} {f\left( {\alpha \left( q \right)} \right) = \alpha \left( q \right)q - \tau \left( q \right)} \\ \end{array}$$

Based on the multifractal analysis methodology described by Xie et al. (2010), the parameters of the multifractal spectrum are calculated to quantify the heterogeneity of the pore structure^60^. The width of the singularity spectrum ($\Delta \alpha$) and the difference in the spectrum function ($\Delta f\left( \alpha \right)$) are defined as:

13 $$\begin{array}{*{20}c} {\Delta \alpha = \alpha_{\max } - \alpha_{\min } } \\ \end{array}$$

14 $$\begin{array}{*{20}c} {\Delta f\left( \alpha \right) = f\left( {\alpha_{\max } } \right) - f\left( {\alpha_{\min } } \right)} \\ \end{array}$$

where $\alpha_{\max }$ and $\alpha_{\min }$ correspond to the maximum and minimum singularity exponents obtained within the range of moment *q*. A larger $\Delta \alpha$ indicates a more inhomogeneous distribution pattern and stronger multifractality.

To further characterize the symmetry of the spectrum, the singularity exponent corresponding to the maximum value of the spectrum function $f\left( \alpha \right)$ is defined as $\alpha_{0}$ (i.e., when $f\left( \alpha \right) = f_{\max }$, $\alpha = \alpha_{0}$). The spectral width is then divided into the left spectral width ($\Delta \alpha_{L}$) and the right spectral width ($\Delta \alpha_{R}$):

15 $$\begin{array}{*{20}c} {\Delta \alpha_{L} = \alpha_{0} - \alpha_{\min } } \\ \end{array}$$

16 $$\begin{array}{*{20}c} {\Delta \alpha_{R} = \alpha_{\max } - \alpha_{0} } \\ \end{array}$$

The asymmetry index (*R*) is subsequently calculated to describe the skewness of the multifractal spectrum:

17 $$\begin{array}{*{20}c} {R = \frac{{\Delta \alpha_{L} - \Delta \alpha_{R} }}{{\Delta \alpha_{L} + \Delta \alpha_{R} }}} \\ \end{array}$$

In expressions (12)–(17), $\Delta \alpha$ is the width of the spectral function opening, $\Delta f\left( \alpha \right)$ represents the range of the spectral function $f\left( \alpha \right)$, $\Delta \alpha_{L}$ represents the left spectral width, $\Delta \alpha_{R}$ represents the right spectral width, and *R* is the asymmetry index of the spectral function^42,60^.

## Samples and experiments

### Pore characteristics

Previous studies have reported that the Taiyuan Formation limestone reservoir in the study area is generally characterized by low porosity and low permeability, with porosity ranging from 0.5% to 4.0%, with an average of 2.1% and samples with porosity greater than 1% account for 56.1%. The average permeability is $0.22\times {10}^{-3}$ µm^2^, and samples with permeability greater than $0.1\times {10}^{-3}$ µm^2^ account for 43.1%^9,12^. As the Taiyuan Formation limestone belongs to low-porosity and low-permeability reservoirs, fractures play a crucial role; they serve as seepage channels for natural gas and improve the storage performance of the limestone, which is significant for determining whether it can become an effective reservoir.

**Fig. 2:**
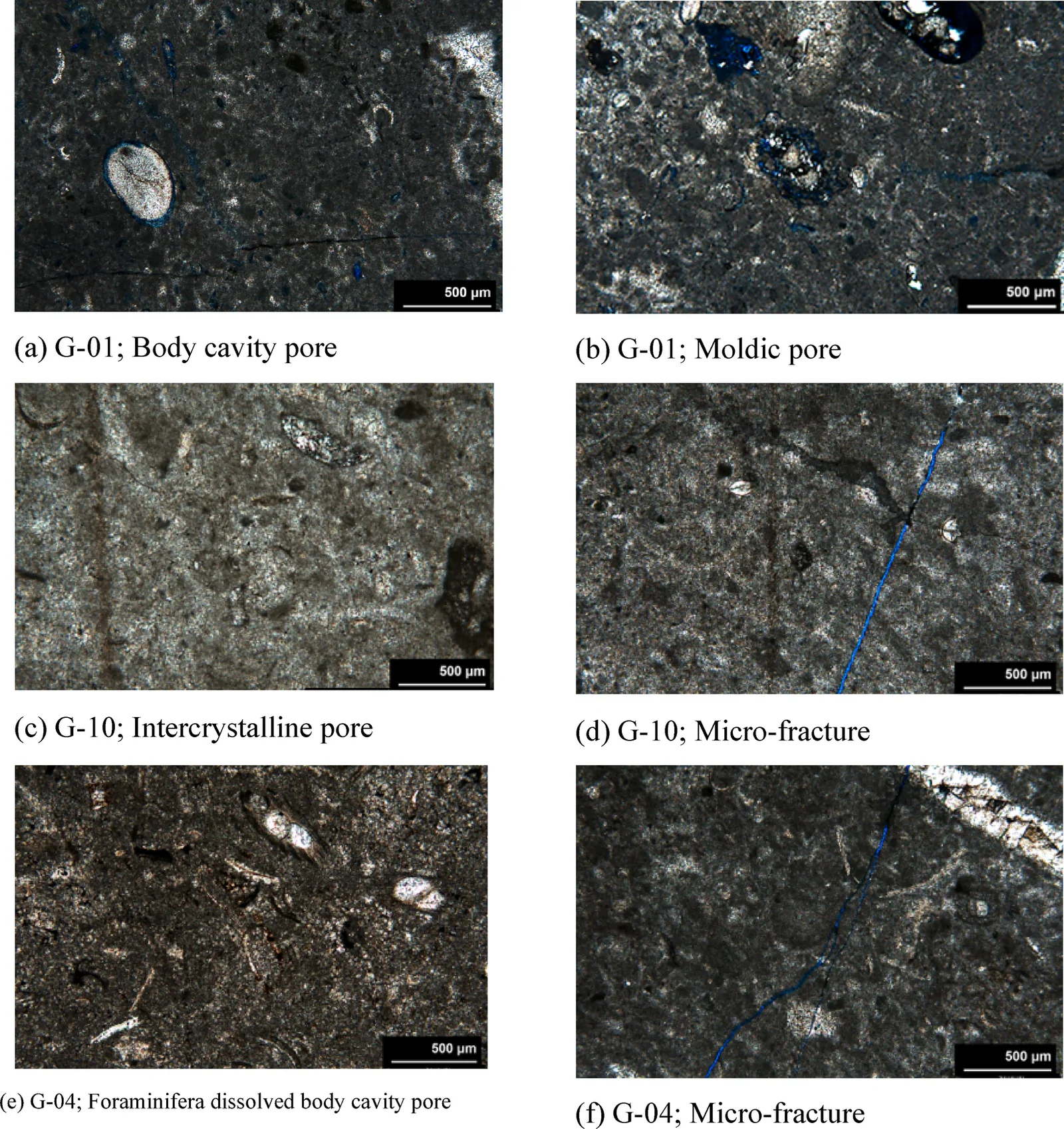
Types of limestone reservoir space in Taiyuan Formation; (**a**) Residual biological body-cavity pore; (**b**) moldic dissolution pore; (**c**) intercrystalline pore; (**d**, **f**) micro-fractures; (**e**) dissolved foraminiferal body-cavity pore.

The reservoir space types of the Taiyuan Formation in the study area mainly include secondary dissolution pores, intercrystalline pores, and micro-fractures. The core samples are mostly bioclastic limestone, gray to grayish-brown, with massive structures, and locally visible millimeter-scale vugs. Microscopic observation shows that the rocks are rich in bioclasts such as foraminifera, bryozoans, and ostracods. Cementation was significant during the depositional period, and dark micrite envelopes developed on grain edges, forming a boundstone texture (Fig. 2).

Primary pores mainly include partially preserved body cavity pores (Fig. 2a, c) and intercrystalline pores (Fig. 2c). Reservoir spaces created by karstification are considered the most important storage spaces in the Taiyuan Formation limestone^12^. Dissolution caused the bioclastic particles to dissolve, forming moldic pores that retain their original shapes (Fig. 2b), which effectively improved reservoir performance.

Multiple micro-fractures are visible under the microscope, usually with widths between 2 and 5 µm, extending from several millimeters to over ten millimeters. These micro-fractures not only possess certain storage capacity themselves but, more importantly, effectively connect a large number of dissolution pores or matrix pores (Fig. 2d, f), greatly enhancing the connectivity of the pore system.

### Pore structure

A total of 10 core samples were collected from the Taiyuan Formation in the Ordos Basin. The samples were selected to cover the main reservoir lithologies and pore-structure types observed in cores and thin sections, including bioclastic limestone and algal/boundstone-related limestone with different degrees of dissolution, cementation, and micro-fracture development. Thin-section observations show that the main reservoir spaces include secondary dissolution pores, moldic pores, intercrystalline pores, residual body-cavity pores, and micro-fractures (Fig. 2). Mercury injection experiments were conducted on all samples, in accordance with the Petroleum Industry Standards of China (SY/T 6385-1999 and SY/T 5346-2005).

The entry pressure of the Taiyuan Formation limestone reservoir mainly ranges between 0.7 and 1.8 kPa. The median pore throat radius varies widely, mainly between 0.1 and 10.0 µm, indicating poor pore throat sorting. The mercury withdrawal efficiency is relatively high, and the overall characteristics are those of medium pores and fine throats. The dissolution pore-throat structures of algal bound limestone and bioclastic silty limestone are generally better. Constant-rate mercury injection experiments indicate that the limestone throat radii are mainly between 0.1 and 0.5 µm, with throat radii between 0.25 and 0.35 µm playing a major role in permeability. Reservoir pores are mainly between 10 and 50 µm, with a pore-to-throat ratio of 100 to 300. FIB-SEM analysis shows that while throat radii in the limestone reservoir do not differ significantly, their quantity is key to influencing the reservoir’s seepage capability^12^.

Based on pore throat parameters obtained from mercury injection experiments, All samples exhibited extremely low displacement pressures, ranging from 0.7 kPa to 1.8 kPa, indicating the widespread presence of easily connected coarse pore throats or micro-fractures in the reservoir. The ${r}_{apex}$ value represents the pore throat size corresponding to the peak of the pore throat distribution curve, which is the radius of the primary pore throats controlling seepage capability. The ${r}_{apex}$ values of the samples are all in the range of hundreds of micrometers, from 356.24 µm to 480.65 µm.

**Fig. 3:**
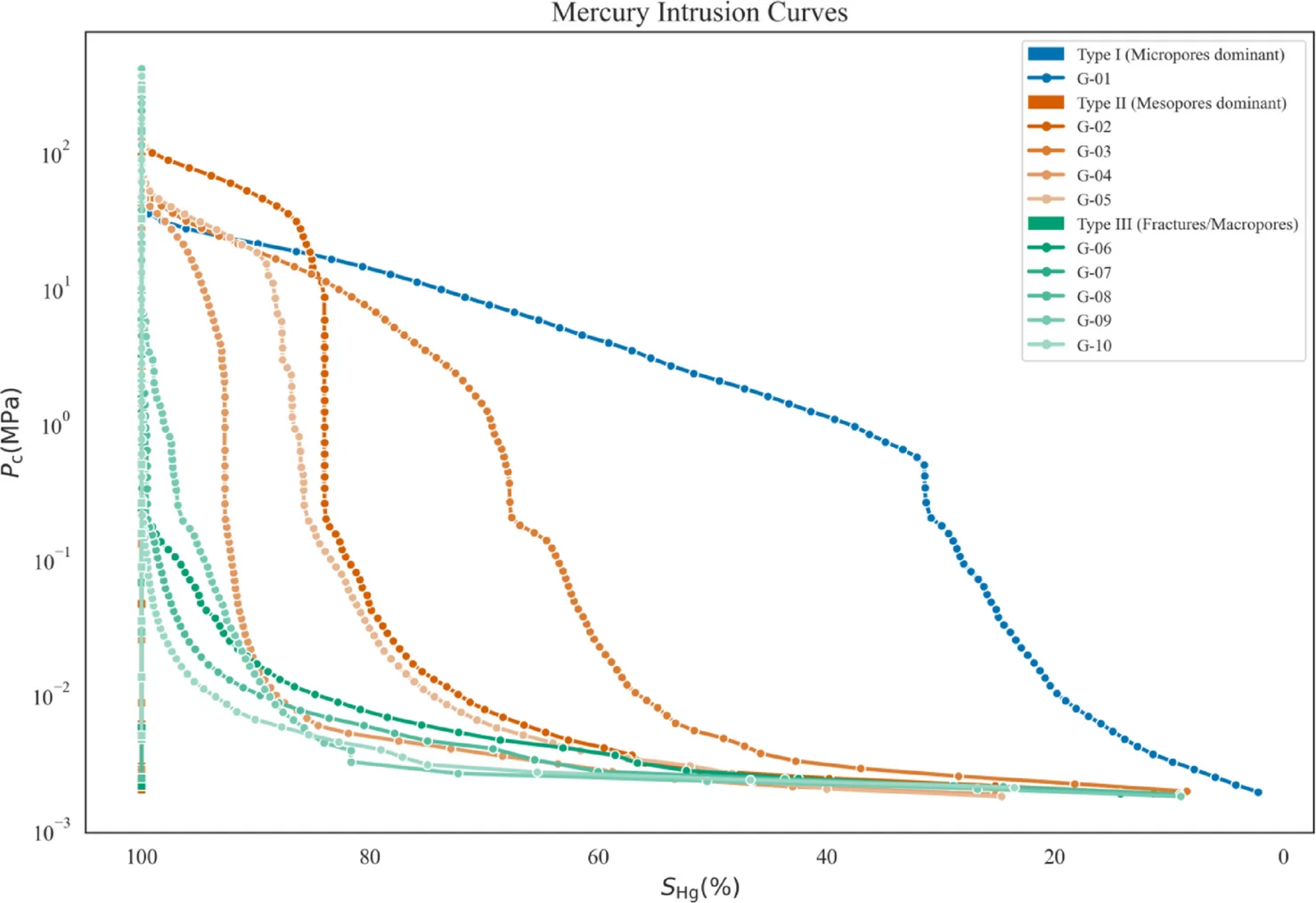
Mercury intrusion curves.

In contrast to the universally low displacement pressures, the microscopic pore throats of the samples show significant differentiation. Porosity varies drastically, from a minimum of 1.85% to 18.41%, a span of nearly 10 times. In terms of pore throat distribution, the median pore throat radius (${r}_{50}$) ranges from 330.33 µm to 1666.97 µm, and the sorting coefficient ranges from 0.7179 to 5.2694, also showing a large span. Furthermore, regarding distribution morphology, sample G-07 has a skewness coefficient of -0.4738, presenting a moderate negative skew, indicating a tendency for the pore throat distribution curve to trail towards small pore throats (Fig. 3).

Based on the morphological characteristics of pore throat distribution and mercury intrusion curves, the reservoirs can be classified into three types:

- *Type I* represents micro-pore throat samples. The curve lacks a distinct mercury intrusion plateau, is gentle in morphology, extends over a long range, and spans multiple orders of magnitude in pressure. The pore throat radius distribution range is extremely wide, and sorting is very poor, with a sorting coefficient as high as 4.8839. Quantitatively, this is characterized by an extremely high sorting coefficient; such reservoirs are dominated by micro-pore throats with poor connectivity.
- *Type II* represents bimodal samples. Their mercury intrusion curves exhibit “step-like” or “dual-plateau” characteristics, with medium displacement pressures between 1.4 and 1.6 kPa. This reflects a bimodal pore throat radius distribution with moderate sorting; sorting coefficients are between 2.3 and 5.3. These samples indicate the coexistence of at least two pore throat systems of different scales, with moderate pore throat connectivity.
- *Type III* represents Fractures/Macropores samples. Their displacement pressures are relatively higher, between 1.6 and 1.8 kPa, but they possess a long and flat plateau, indicating that mercury intrusion pressure is concentrated within a very narrow range. This reflects a narrow range of pore throat radius distribution, high concentration, and excellent sorting. Quantitatively, they exhibit extremely low sorting coefficients; samples G-06, G-07, G-08, G-09, and G-10 have sorting coefficients between 0.7179 and 1.2979, confirming this feature. Such reservoirs are dominated by well-sorted coarse pore throats with good connectivity (Table 1).

**Table 1:** Parameters of the pore throat structure.

Sample No	Depth	Entry pressure	Porosity	Sorting coefficient	Skewness	Kurtosis	r_35_	P_35_	r_50_	P_50_	r_apex_
	m	kPa	%		Skp	Kp	μm	MPa	μm	MPa	
G-01	3403.00	0.7	3.5201	4.8839	-0.4738	0.6862	1.6314	0.7396	0.5729	2.1318	378.01
G-02	2544.00	1.6	1.8476	5.2694	0.9317	2.2404	535.5766	0.0022	445.3029	0.0028	472.33
G-03	3244.16	0.7	3.0592	5.2429	0.7888	0.5488	445.7783	0.0027	244.1914	0.005	378.01
G-04	3057.00	1.5	10.1501	2.3506	0.7484	3.9813	616.1546	0.002	544.1556	0.0022	356.243
G-05	2337.00	1.4	4.1982	3.7117	0.8262	2.396	555.7383	0.0024	429.8483	0.0028	480.648
G-06	2849.00	1.6	2.4772	1.2979	0.7179	1.332	546.1524	0.0022	461.2122	0.0027	472.33
G-07	2849.00	1.7	3.9941	1.2588	0.8643	1.9126	330.3342	0.0037	742.5809	0.0012	476.355
G-08	3274.00	1.7	6.0597	1.0072	0.6421	1.2978	545.3912	0.0022	490.0786	0.0025	467.47
G-09	3465.00	1.7	5.4564	1.2206	0.7727	4.5082	1666.971	0.0082	682.5198	0.0009	467.47
G-10	2337.00	1.8	18.4082	0.7179	0.5714	1.9126	572.5762	0.0021	504.4273	0.0024	480.648

## Results and discussion

### Single fractal characteristics

**Fig. 4:**
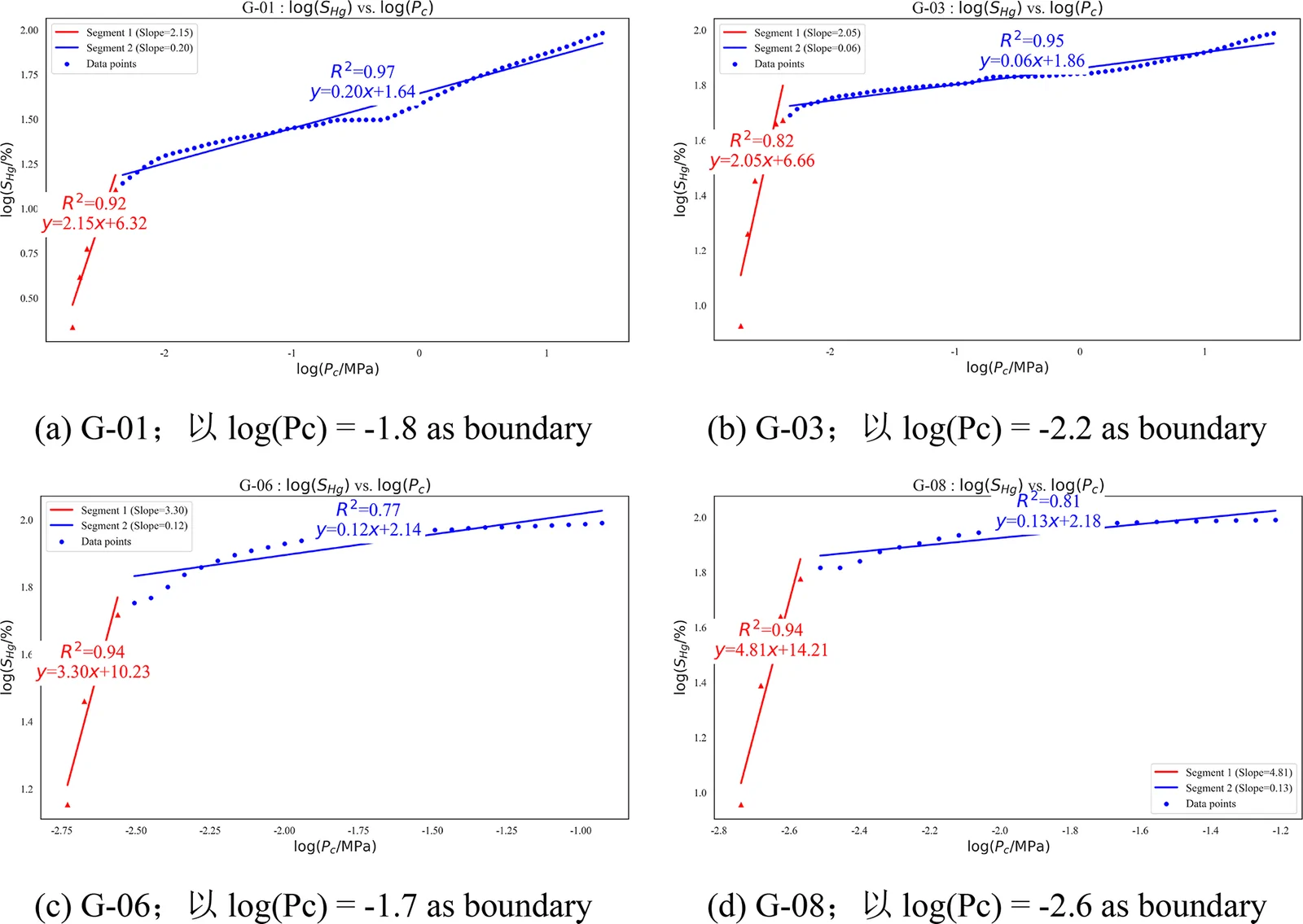
Plots of log(S_Hg_) vs log(Pc) from the mercury intrusion curve of G-01, G-03, G-06, G-08.

To quantitatively evaluate the complexity of the pore structure, based on the Li (2010) model, this study constructed scatter plots of log capillary pressure ($\mathrm{l}\mathrm{o}\mathrm{g}{P}_{c}$) versus log mercury saturation ($\mathrm{l}\mathrm{o}\mathrm{g}{S}_{Hg}$) for all 10 tight carbonate rock samples. A linear regression method was used for segmented fitting of the point distribution (Fig. 4). The corresponding fractal dimension *D* was calculated from the slope of the fitted line to quantitatively characterize the fractal distribution features of the pore structure (Table 2). Overall, the correlation coefficients ${R}^{2}$ of the fitted segments range from 0.73 to 0.96, indicating that the pore systems of the tight carbonate rocks in the study area possess good fractal characteristics.

**Table 2:** Parameters of the fractal dimension obtained from the 10 samples.

Sample no	D_1_	Correlation coefficient R^2^	D_2_	Correlation coefficient R^2^
G-01	5.1525	0.9226	3.1959	0.9673
G-02	4.5984	0.7354	3.0216	0.7304
G-03	5.0459	0.8205	3.0586	0.9514
G-04	3.7556	0.7946	3.0085	0.8258
G-05	3.6458	0.8117	3.0226	0.8758
G-06	6.301	0.9355	3.1233	0.7737
G-07	3.5438	0.8885	3.0358	0.9376
G-08	7.8128	0.944	3.1259	0.8068
G-09	8.2963	0.9456	3.029	0.9024
G-10	5.9644	0.9055	3.1158	0.8742

From the plots, it can be clearly observed that the $\mathrm{l}\mathrm{o}\mathrm{g}\left({S}_{Hg}\right)$ vs. $\mathrm{l}\mathrm{o}\mathrm{g}\left({P}_{c}\right)$ curve exhibits an inflection point within the range of logarithmic capillary pressure equal to − 1.7 to − 2.6, dividing into segments with different slopes. According to the capillary model, this separation interval roughly corresponds to ideal capillaries with radii *r≈* 36.5–292.0 µm. Therefore, the left segment provides information about pores with very large radii, such as micro-fractures or super-large pores, while the right segment represents pores with radii less than *36.5* µm.

To improve reproducibility, candidate segmentation points near the inflection interval were tested using two-segment linear fitting. The final boundary was selected by considering the combined fitting quality of the two linear segments together with the geological meaning of the corresponding pore-throat interval, rather than by visual inspection alone.

Using Eq. (1), the number of segments for each sample was calculated. The ${D}_{1}$ values vary significantly between 3.54 and 8.30. Samples G-08 and G-09 have anomalously high ${D}_{1}$ values of 7.8128 and 8.2963, respectively, indicating more intensive development of micro-fractures^76^. Conversely, G-05 and G-07 have the lowest ${D}_{1}$ values, only 3.6458 and 3.5438, representing relatively simple macroscopic pore structures. In sharp contrast to ${D}_{1}$, ${D}_{2}$ values are concentrated in a very narrow interval, with all values between 3.00 and 3.19. The significant difference between the two indicates that large pores and small pores belong to different fractal systems. It should be noted that ${D}_{1}$ is not a true geometric fractal dimension of the pore space, but an apparent index derived from the low-pressure segment of the MIP curve. Therefore, ${D}_{1}$ values higher than the theoretical range mainly indicate the influence of large pore throats, micro-fractures, and complex seepage pathways. Similar abnormal ${D}_{1}$ values have been reported in MIP-based fractal studies and were attributed to the influence of microfractures and limitations of single-fractal models^76,78^.

### Multifractal characteristics

Although single fractal analysis can effectively distinguish the average heterogeneity of seepage pores and matrix pores, this method essentially simplifies the pore system into a few discrete subsets, each with a single fractal characteristic. However, the pore structure of tight reservoirs is extremely complex, and its heterogeneity may not be a simple two-stage distribution but rather presents continuous variation of scaling indices across different scales.

To more finely characterize this continuous, multi-scale heterogeneity, this paper further introduces multifractal analysis^60^. Multifractal analysis does not calculate a single fractal dimension but describes the entire statistical moment spectrum of pore distribution through the generalized dimension spectrum $D_{q}$ and the $f\left( \alpha \right)$ singularity spectrum. The pore volume distribution function *dv/dłog D* from MIP is selected to construct the partition function, and then the multifractal method is used to analyze the pore distribution characteristics of the samples.

**Fig. 5:**
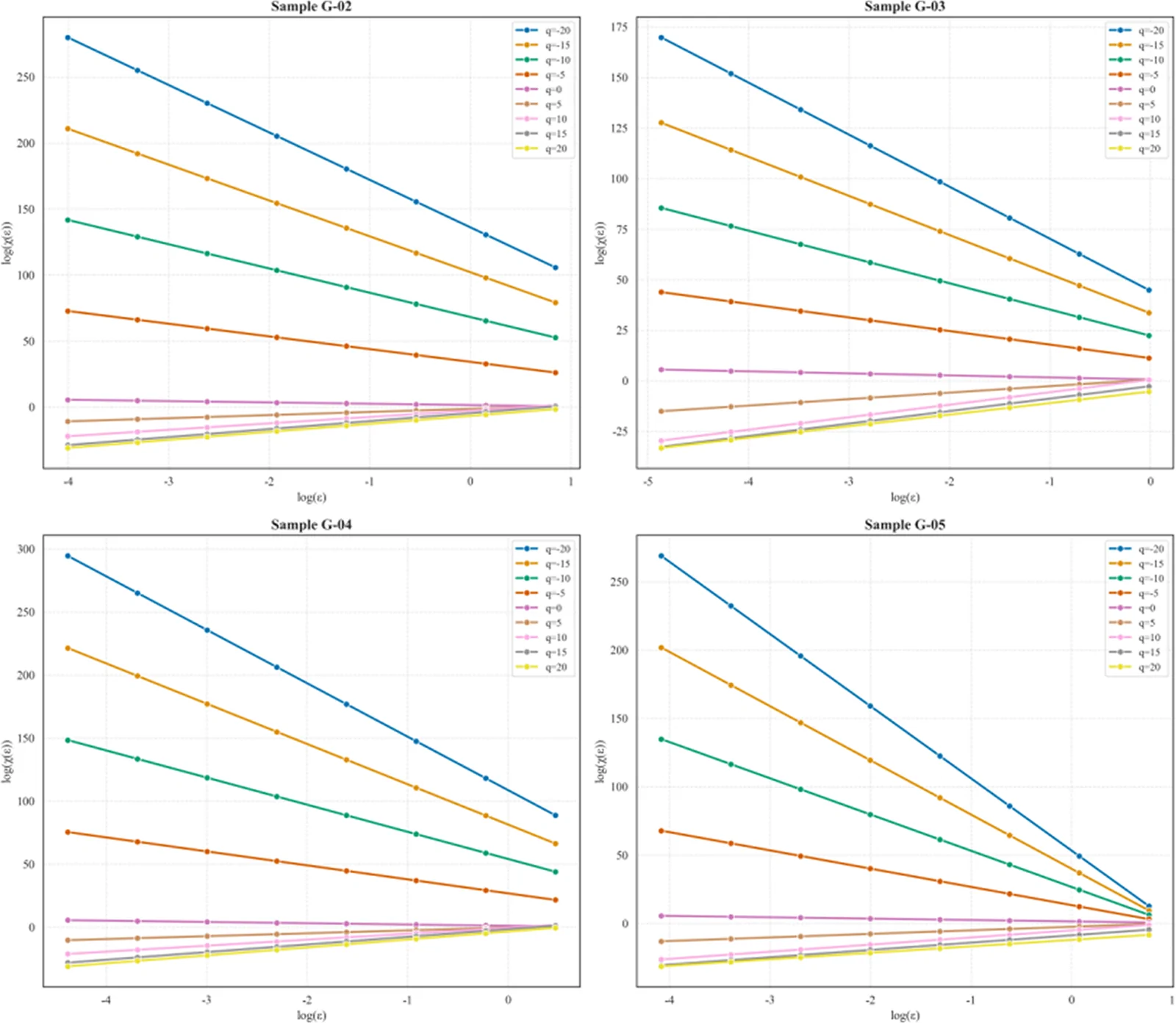
Plots of μ(ε) versus box size ε, for the pore size distribution.

To verify the applicability of this method to the samples in this study, we first tested the linear relationship between the partition function $\mathrm{l}\mathrm{o}\mathrm{g}\left(\chi \left(\varepsilon \right)\right)$ and $\mathrm{l}\mathrm{o}\mathrm{g}\left(\varepsilon \right)$. When *q* ranges from − 20 to 20, the relationship between log(*ɛ*) and log($\chi (\varepsilon )$) is generally linear for most samples and most moment orders, indicating that the moment method is applicable to the present dataset. According to formula (5), the slope *k* of the double-logarithmic fit of $\mathrm{l}\mathrm{o}\mathrm{g}\left(\varepsilon \right)$ and $\mathrm{l}\mathrm{o}\mathrm{g}\left(\chi \left(\varepsilon \right)\right)$ is the mass exponent $\tau \left(q\right)$. Figure 5 shows the partition functions of four samples at different *q* values in double-logarithmic coordinates show that as *q* increases from − 20 to 20, the corresponding $\tau \left(q\right)$ increases, indicating that the samples possess multifractal characteristics in spatial distribution.

The same moment-order range was applied consistently to all samples to ensure comparability within the dataset. Negative q values emphasize low-probability pore-size intervals, whereas positive q values emphasize high-probability intervals; therefore, the selected range allows both sparse and dominant pore-size domains to be characterized. Similar moment-order ranges and multifractal interpretations have been used in previous studies of mercury-intrusion pore-size distributions and reservoir pore heterogeneity^79^.

**Fig. 6:**
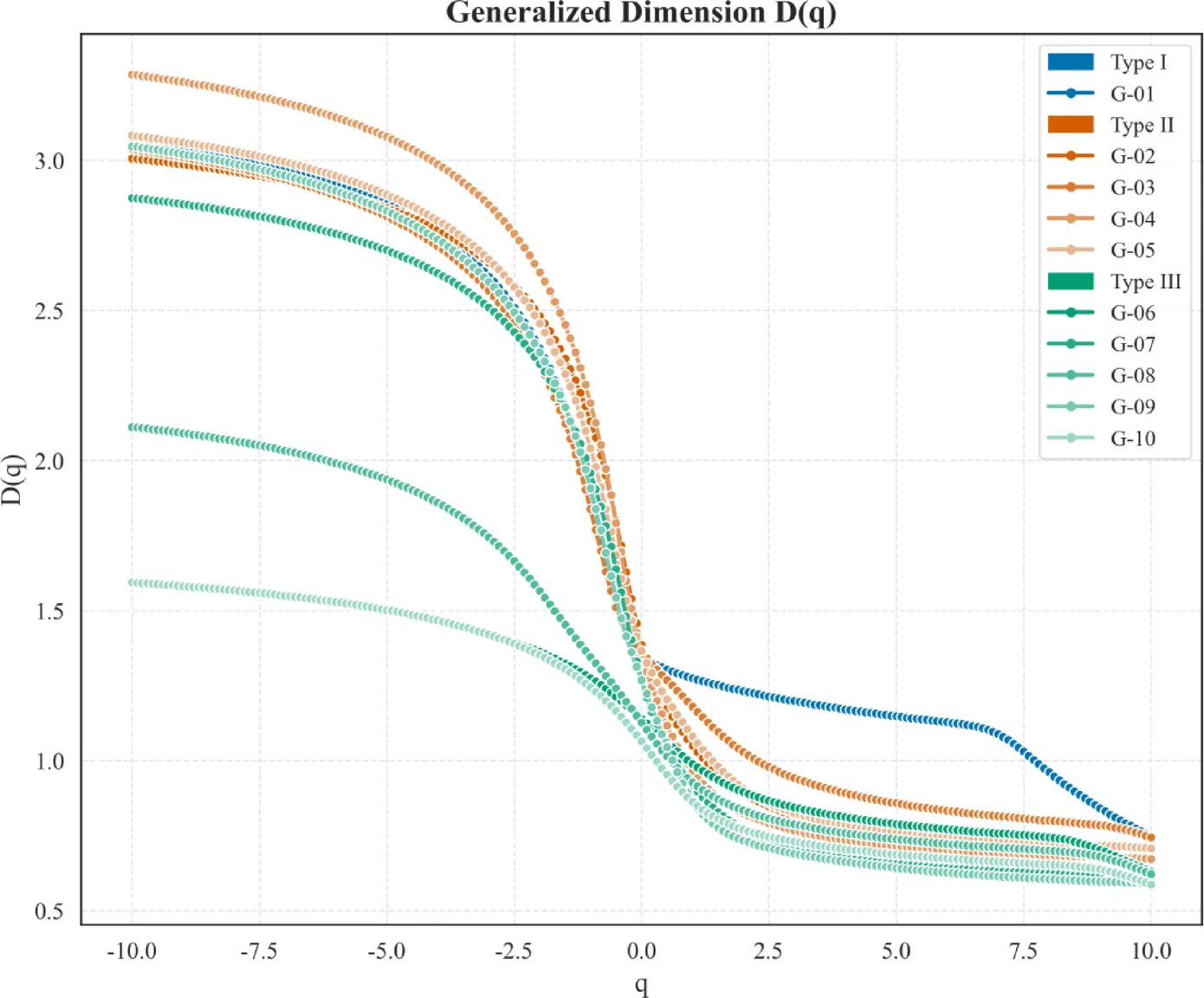
Plot of the multifractal generalized dimension D(q) versus the moment order q.

The generalized dimension spectrum is one of the core tools for characterizing multifractal features^60^. According to formula (6), the generalized dimension $D\left(q\right)$ is calculated within the range of $-10\le q\le 10$. The relationship between the multifractal generalized dimension $D\left(q\right)$ and the moment order *q* is shown in the Fig. 6, and the corresponding ${R}^{2}$ are listed in the Table 3.

**Table 3:** Correlation determination R^2^ of the fitting lines between log(ε) and log(χ(ε)).

q	Correlation coefficient R^2^									
	G-01	G-02	G-03	G-04	G-05	G-06	G-07	G-08	G-09	G-10
− 20	1	1	0.95	0.97	0.96	1	1	1	1	1
− 15	1	1	0.95	0.97	0.96	1	1	1	1	1
− 10	1	1	0.96	0.97	0.97	1	1	1	1	1
− 5	1	1	0.97	0.98	0.98	1	1	1	1	1
0	1	1	1	1	1	1	1	1	1	1
5	1	0.98	0.99	0.98	0.98	0.99	0.97	0.99	0.96	0.96
10	0.86	0.98	0.99	0.98	0.97	0.99	0.97	0.99	0.96	0.96
15	0.47	0.96	0.91	0.98	0.98	0.96	0.98	0.98	0.96	0.96
20	0.43	0.89	0.82	0.94	0.9	0.89	0.94	0.95	0.95	0.93

The relationship between generalized fractal dimension $D\left(q\right)$ and moment order *q* reveals that according to multifractal theory, the morphological characteristics of the $D\left(q\right)$ curve can characterize the heterogeneity of the pore structure^80^. For a completely homogeneous fractal, the $D\left(q\right)$ curve appears as a straight line; for a heterogeneous fractal, the curve exhibits a certain width and curvature, where greater curvature indicates stronger heterogeneity of the sample’s pore distribution.

Figure 6 shows that all samples exhibit a certain degree of curvature and present heterogeneity. Most samples, such as G-07, G-02, and G-08, have very similar and highly clustered $D\left(q\right)$ curve shapes. In sharp contrast, the $D\left(q\right)$ curve shapes for samples G-01 and G-10 are significantly flatter, with curvature significantly smaller than all other samples, suggesting that the pore distribution of G-01 and G-10 is relatively more homogeneous.

Capacity dimension $D\left(0\right)$, information dimension $D\left(1\right)$, and correlation dimension $D\left(2\right)$ are listed in the Table 4.

**Table 4:** Multifractal dimension parameters obtained from the 10 samples.

Sample	D(0)	D(1)	D(2)	D(0)/D(1)	D_min	D_max	ΔD
G-01	1.35	1.27	1.23	1.06	0.75	3.08	2.33
G-02	1.39	1.05	0.89	1.32	0.67	3.01	2.34
G-03	1.36	1.18	1.03	1.15	0.74	3.04	2.29
G-04	1.36	0.96	0.82	1.41	0.67	3.29	2.61
G-05	1.37	1.08	0.91	1.26	0.71	3.08	2.38
G-06	1.14	0.99	0.9	1.15	0.63	1.59	0.96
G-07	1.27	0.92	0.77	1.38	0.61	2.88	2.26
G-08	1.13	0.93	0.83	1.21	0.62	2.11	1.49
G-09	1.27	0.87	0.73	1.46	0.59	3.05	2.46
G-10	1.06	0.86	0.77	1.23	0.59	1.59	1.01

Capacity Dimension $D\left(0\right)$ reflects the breadth of pore size distribution. Among all samples, G-02 and G-05 have the relatively largest $D\left(0\right)$ values (1.39 and 1.37), indicating a wide range of pore size distribution with diverse pore types and large size spans. In contrast, G-10 and G-06 have the smallest $D\left(0\right)$ values (1.06 and 1.14), indicating narrower pore size distributions and more singular pore types.

Information Dimension $D\left(1\right)$ reflects the concentration and heterogeneity of pore size distribution. Sample G-01 has the highest $D\left(1\right)$ value of 1.27, indicating its pore distribution spans a wide size range with the strongest inhomogeneity. Conversely, G-10 and G-09 have the lowest $D\left(1\right)$ values (0.86 and 0.87), indicating more uniform and concentrated pore distributions.

Ratio $D\left(0\right)/D\left(1\right)$ shows the discreteness of pore size distribution. When this ratio is close to 1, new pores are concentrated in dense areas. Sample G-10 has a ratio of 1.06, closest to 1, indicating pores are mostly concentrated as micropores. Samples G-09 and G-04 have the largest ratios (1.46 and 1.41), indicating discrete pore size distributions biased towards sparse regions.

**Fig. 7:**
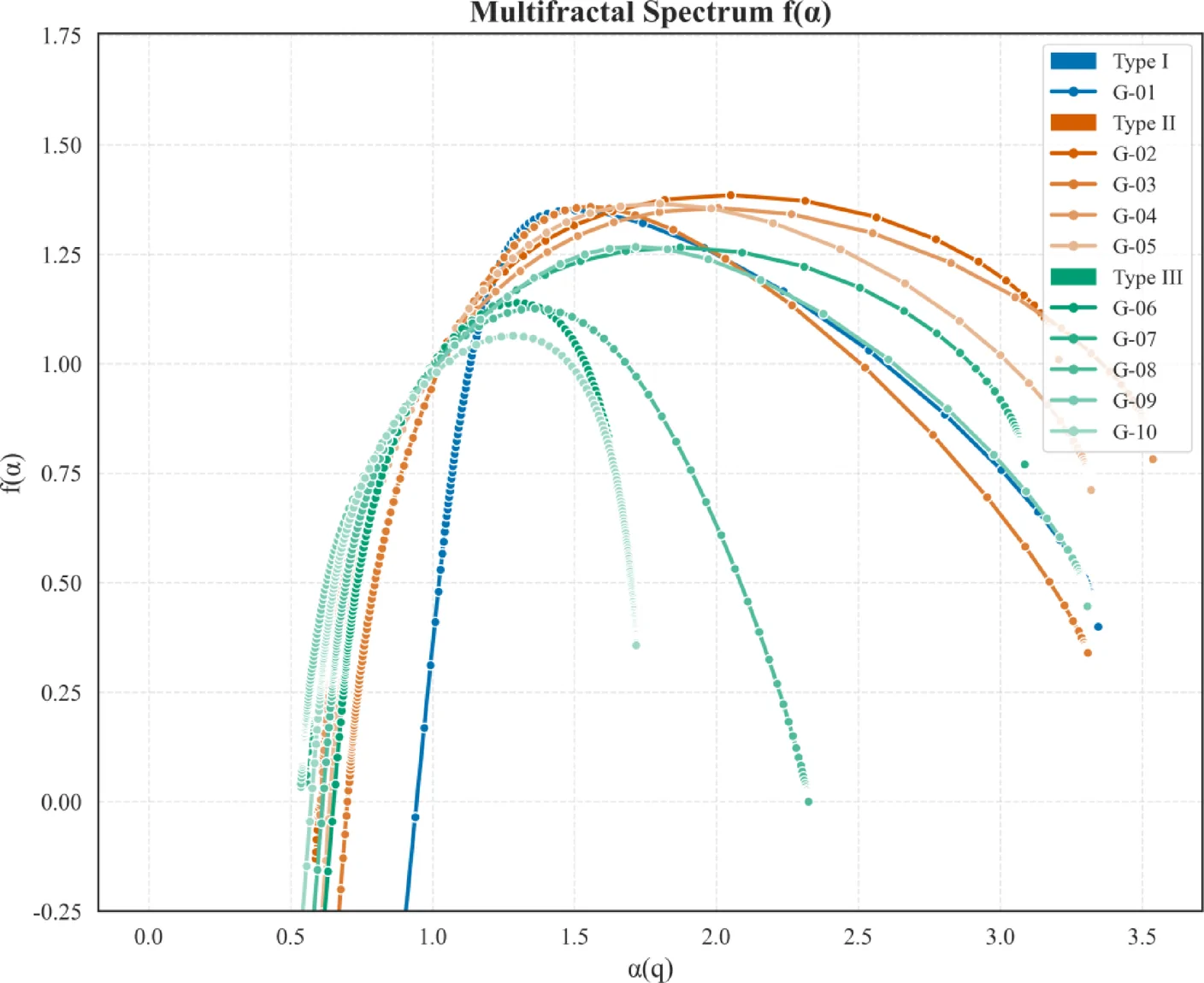
Plot of multifractal spectrum *f(α )* versus the singularity strength *α*.

Multifractal Spectrum Width *Δ D* is the difference between $D_{\max }$ and $D_{\min }$, acting as a direct indicator of heterogeneity; larger *Δ D* implies stronger heterogeneity. Sample G-04 has the largest *Δ D* (2.61), and G-09 and G-01 are also relatively high, indicating robust and significant heterogeneity. In contrast, G-06 and G-10 have the smallest *Δ D* values (0.96 and 1.01), indicating relatively weak heterogeneity and more uniform pore structures (Fig. 7) (Table 5).

**Table 5:** Multifractal spectrum parameters obtained from the 10 samples.

Well	$\Delta \alpha$	*α*	${\alpha}_{L}$	${\alpha}_{R}$	*Δ f*	*R*	Type
G-01	3.345	1.501	1.501	1.845	8.079	− 0.103	Type I
G-02	2.619	2.05	1.463	1.156	1.515	0.117	Type II
G-03	3.098	1.556	1.347	1.751	5.964	− 0.13	
G-04	2.934	2.006	1.404	1.53	1.386	− 0.043	
G-05	2.753	1.8	1.234	1.52	2.061	− 0.104	
G-06	1.704	1.297	1.287	0.416	6.724	0.512	Type III
G-07	2.531	1.872	1.318	1.213	1.222	0.041	
G-08	2.283	1.361	1.32	0.963	6.325	0.157	
G-09	2.772	1.716	1.18	1.591	1.233	− 0.148	
G-10	1.666	1.283	1.231	0.434	5.835	0.478	

$\Delta \alpha$ is the width of the multifractal spectrum (difference between $\alpha_{\max }$ and $\alpha_{\min }$), representing the singularity of the pore structure distribution; In general, a larger $\Delta \alpha$ may indicate a wider range of local singularity strengths and stronger pore-structure heterogeneity^81,82^. The $\Delta \alpha$ values for the 10 samples range from 1.666 to 3.345. Sample G-01 has the maximum $\Delta \alpha$ (3.345), indicating the strongest pore structure heterogeneity. Samples G-03 and G-04 also have relatively high values (3.098 and 2.934), showing strong heterogeneous characteristics. In contrast, G-10 has the smallest $\Delta \alpha$ (1.666), and G-06 is also low (1.704), confirming that their pore system heterogeneity is relatively weakest and distribution is statistically most uniform.

*R* is the asymmetry index and describes the relative skewness of the spectrum, capable of characterizing the relative degree of differentiation between large and small pores^13,35^. When *R* is negative, the multifractal spectrum has a wider right side, indicating rock singularity is dominated by smaller pores. When *R* is positive, the spectrum has a wider left side, indicating singularity is dominated by larger pores; the larger the absolute value of *R*, the greater the singularity difference between large and small pores^45,60^.

The *R* values for the 10 samples range from − 0.148 to 0.512, showing significant variation in pore structure control mechanisms among samples. Sample G-06 has the maximum *R* value (0.512), and G-10 is also relatively high (0.478), indicating that their pore systems are strongly dominated by large pores or fracture-related seepage channels. Samples G-08 and G-02 also exhibit positive *R* values (0.157 and 0.117), suggesting a certain degree of large-pore control.

In contrast, samples such as G-03, G-05, and G-01 show negative *R* values (− 0.13, − 0.104, and − 0.103), indicating that their heterogeneity is mainly governed by smaller pores. Among them, G-09 has the most negative *R* value (− 0.148), reflecting the strongest dominance of micropores. Overall, the variation of *R* reveals that the pore systems transition from small-pore-dominated storage structures to large-pore- or fracture-dominated seepage systems

**Fig. 8:**
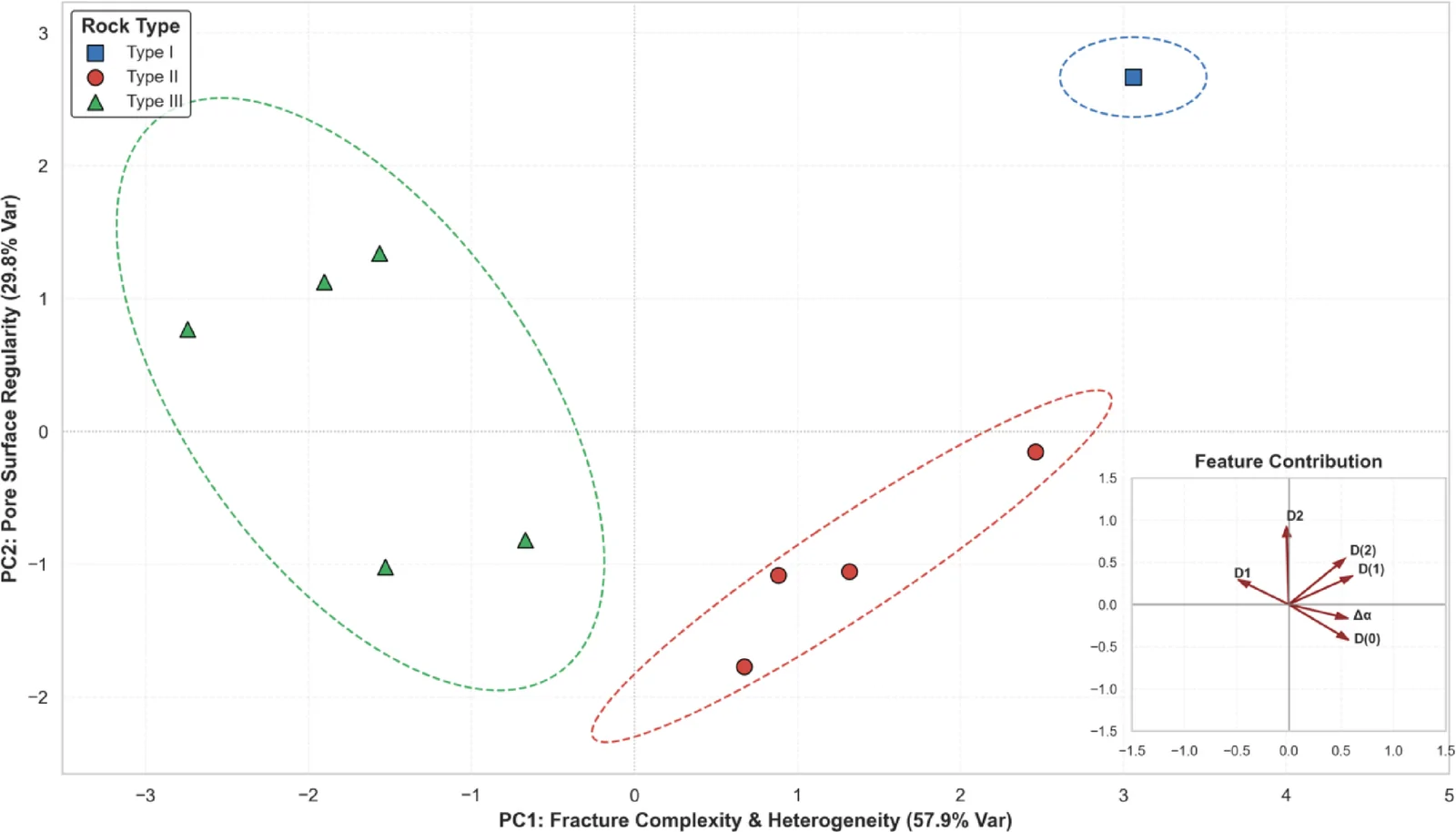
Classification of pore structure types based on multifractal parameters.

To quantitatively characterize the heterogeneity and complexity of the reservoir pore structure, six representative multifractal parameters were selected as feature vectors for clustering analysis (Fig. 8), including the generalized fractal dimensions (*D(0)*, *D(1)*, *D(2)*), the single fractal dimensions (${D}_{1}$, ${D}_{2}$), and the width of the multifractal spectrum ($\Delta \alpha$). These parameters were chosen for their distinct physical significance in describing pore connectivity, irregularity, and the span of pore size distribution^26,60,70^.

A hybrid analytical approach combining Principal Component Analysis (PCA) and K-Means clustering was employed to classify the samples. First, the raw data underwent Z-score normalization to eliminate dimensional discrepancies between parameters. PCA was then applied to project the high-dimensional feature space into a lower-dimensional subspace, extracting orthogonal principal components that capture the maximum variance while minimizing noise^83^. Subsequently, unsupervised K-Means clustering (with *k=3*) was performed on the principal components to identify distinct rock types^84^, using 95% confidence ellipses to delineate the distribution boundaries of each cluster.

To evaluate the internal consistency of the clustering result, the silhouette coefficient was calculated for the PCA–K-means classification^85^. For k = 3, the mean silhouette coefficient is 0.47, suggesting a moderate degree of separation among the three pore-structure classes. The choice of k = 3 is also consistent with the three pore-throat types identified from mercury-intrusion curve morphology. However, considering the limited number of samples, the clustering result should be interpreted as an exploratory pore-structure classification rather than a fully validated predictive model (Table 6).

**Table 6:** KMO and Bartlett’ Test.

(KMO) Measure of sampling adequacy	–	0.607
Bartlett’s Test of Sphericity	Approximate Chi-Square	61.866
	Degrees of freedom	15
	Significance/*p* value	< 0.001

The proposed method provides a reasonable exploratory classification, with the first two principal components (PC1 and PC2) accounting for a cumulative variance of 87.8% (57.9% and 29.8%, respectively) (Fig. 8, Table 7). This high explanation ratio indicates that the 2D projection preserves the vast majority of the original structural information. Physically, PC1 is primarily driven by ${D}_{1}$, ${D}_{2}$, and $\Delta \alpha$, characterizing the fracture complexity and overall heterogeneity, whereas PC2 is dominated by the single fractal dimension, reflecting pore surface regularity. The resulting clusters (Types I, II, and III) exhibit distinct boundaries and correlate well with petrophysical properties, supporting the use of the classification as a preliminary reservoir-quality evaluation framework.

**Table 7:** Total variance explained (principal component analysis).

Initial Eigenvalues				Extraction sums of squared loadings		
Component	Total	Variance	Cumulative	Total	Variance%	Cumulative %
1	3.477	57.946	57.946	3.477	57.946	57.946
2	1.791	29.844	87.790	1.791	29.844	87.790
3	0.589	9.823	97.613			
4	0.112	1.862	99.476			
5	0.028	0.459	99.935			
6	0.004	0.065	100.000			

Type I (Micro-pore throat): Sample G-01 has the strongest heterogeneity (max $\Delta \alpha$). Its *R* value is negative, showing distinct right-skewness. This indicates that while its pore structure is extremely complex, this strong heterogeneity is mainly contributed by smaller matrix pores.

Type II (Bimodal): These also show strong heterogeneity, but sources vary. G-03, G-04, and G-05 are right-skewed (negative *R*), consistent with Type I (small pore dominated). However, G-02 is an exception with a positive *R* (left-skewed), indicating heterogeneity originates from larger pores, distinguishing it structurally from others in the group.

Type III (Fractures/Macropores): Heterogeneity is relatively weak; $\Delta \alpha$ values are generally lower than the first two types. *R* values are generally positive (left-skewed), indicating structural heterogeneity is dominated by large seepage channels. Notably, internal differences are significant: G-06 and G-10 have the weakest heterogeneity but highest absolute *R* values, suggesting that while relatively homogeneous, the dominance of large pores is strongest.

Single fractal dimension calculation relies on specific geometric models: Friesen used the Sierpinski carpet model^51^, Shen assumed a capillary bundle model^86^, and Su established a model considering pore space and tortuosity dimensions^52^. Different models yield different dimensions^87^. However, when micro-fractures develop, ${D}_{1}$ values present “abnormally” high values far exceeding theoretical thresholds; this is because current fractal models assume reservoirs consist only of pores, ignoring micro-fractures. In this study, ${D}_{1}$ values for G-08 and G-09 reached 7.81 and 8.30, respectively. This significant deviation is a sensitive response of the single fractal model to micro-fracture development, not calculation error.

In contrast, multifractal analysis is based on statistical moment methods and does not require presupposing specific pore geometric shapes. Multifractal parameters like $\Delta \alpha$ reflect the breadth and concentration of pore distribution in a statistical sense, rather than the physical dimensions of a specific geometric structure. This explains why G-08, despite having an extremely high ${D}_{1}$ and well-developed fractures, has a moderate multifractal spectrum width $\Delta \alpha$, as its overall pore distribution heterogeneity is not as great as that of G-01.

Cluster analysis in this study shows that combining mercury intrusion parameters with fractal parameters ${D}_{1}$ and $\Delta \alpha$ achieves the most accurate reservoir classification^88^. It is recommended to use the single fractal parameter ${D}_{1}$ as a specific indicator for identifying micro-fracture development and evaluating macroscopic seepage channel complexity, while using multifractal parameters $\Delta \alpha$ and *R* as indicators to measure overall reservoir heterogeneity and pore distribution patterns.

### Implications for reservoir evaluation

Traditional reservoir evaluation often relies on porosity as the primary indicator of storage capacity^89^. However in the Taiyuan Formation limestones, Samples with high porosity do not necessarily exhibit high seepage capacity, primarily due to the complexity of the pore throats^35^. The fractal–multifractal characterization conducted in this study therefore provides additional quantitative descriptors for evaluating pore-structure heterogeneity in tight carbonate reservoirs^90^.

Type I reservoirs are characterized by extremely high multifractal spectrum width ($\Delta \alpha$) and high single fractal dimensions in the small-pore region (${D}_{2}$). Although these samples may exhibit measurable porosity, the high ${D}_{2}$ values indicate an extremely rough and complex pore surface structure. Previous studies have suggested that high fractal dimensions in the matrix domain may be associated with specific surface area, leading to high irreducible water saturation (${S}_{wi}$) and high capillary bound fluid volume^91,92^. The negative asymmetry index (*R<0*) suggests that the heterogeneity is dominated by small, isolated micropores rather than effective flow channels. Consequently, despite the presence of storage space, Type I reservoirs appear less favorable for effective fluid mobility from the perspective of pore-structure characterization^93^.

Type II reservoirs exhibit a bimodal pore size distribution and intermediate fractal parameters, representing a transition zone between the tight matrix and developed seepage channels. These reservoirs possess a certain degree of macroscopic connectivity, as indicated by the emergence of a second mercury intrusion plateau. However, the spectrum width ($\Delta \alpha$) remains relatively high, suggesting significant heterogeneity and the associated connectivity may therefore be less uniform^34,76^.

Type III reservoirs are characterized by a distinct 'left-skewed’ multifractal spectrum (*R>0*) and significantly higher fractal dimensions (${D}_{1}$), indicating the development of complex micro-fracture networks. The positive asymmetry index (*R>0*) is a critical indicator that the pore structure heterogeneity is driven by large pores and micro-fractures^94,95^. This structural feature ensures that fluids can bypass the tight matrix, providing superior permeability. These samples exhibit the moderate to low ${D}_{2}$ values, implying smoother pore surfaces and lower irreducible water saturation, effectively maximizing the volume of mobile hydrocarbons^26^.

These results suggest that reservoir evaluation in tight carbonate limestones should not rely on porosity alone, and that connectivity-related indicators such as ${D}_{1}$, *R*, and $\Delta \alpha$ may provide useful supplementary constraints. Within the present sample set, Type III reservoirs appear to represent the most favorable pore-structure conditions for fluid connectivity, although this interpretation still requires validation using additional petrophysical and production-related data.

Nevertheless, these interpretations are based on a limited number of samples and primarily on mercury-intrusion-derived pore-structure data. Such limitations are common in pore-structure studies of tight reservoirs, where multiscale heterogeneity and complex pore–throat systems often require the integration of multiple analytical techniques and datasets^96,97^. Previous studies have also demonstrated that fractal and multifractal parameters provide effective descriptors of pore heterogeneity and are closely related to fluid flow behavior, although the relationships may vary depending on reservoir conditions^18,60,98^. Therefore, further validation using larger datasets and independent petrophysical, imaging, and production-related evidence is recommended. Despite these limitations, the relationships identified in this study remain internally consistent and are supported by both the observed experimental trends and previously reported pore-structure–seepage correlations. The results therefore provide a reasonable and meaningful basis for reservoir-quality evaluation within the scope of the present dataset.

## Conclusions

In this study, single fractal and multifractal theories were applied to mercury intrusion data to characterize the pore structure heterogeneity of tight carbonate reservoirs. By integrating physical properties with fractal parameters, a pore-structure-based classification framework was developed for the studied samples. The following conclusions can be drawn:

1. The pore systems of the Taiyuan Formation limestones exhibit significant self-similarity, characterized by a segmented single fractal behavior. The fractal dimension ${D}_{1}$ in the low-pressure region varies substantially and serves as a sensitive indicator for the development of micro-fractures and macroscopic seepage channels. In contrast, the fractal dimension ${D}_{2}$ in the high-pressure region remains relatively stable, reflecting the intrinsic structural complexity of the matrix pores.
2. Multifractal analysis provides a more robust statistical description of the overall pore size distribution without relying on specific geometric assumptions. The spectral width $\Delta \alpha$ effectively quantifies the global heterogeneity of the reservoir, revealing that samples dominated by micro-pores possess the strongest heterogeneity. Furthermore, the asymmetry index *R* provides a supplementary indication of whether pore-structure heterogeneity is more strongly associated with large seepage pores or small storage pores within the studied dataset.
3. Based on the pore throat characteristics and fractal parameters, the studied samples are classified into three types. Importantly, Type III reservoirs, characterized by a positive asymmetry index (*R>0*), high low-pressure fractal dimension (${D}_{1}$), and moderate spectrum width ($\Delta \alpha$), appear to represent more favorable pore-structure conditions for fluid connectivity, whereas high ${D}_{2}$ values in matrix-dominated pore systems are associated with less favorable mobility conditions.
4. The integration of single fractal and multifractal analyses offers a superior approach for reservoir characterization compared to using either method alone. The single fractal parameter ${D}_{1}$ is useful for identifying structural differences related to large-pore or fracture development, whereas $\Delta \alpha$ and *R* help describe the overall heterogeneity of the pore system. Therefore, the combination of relatively high ${D}_{1}$, positive *R*, and moderate $\Delta \alpha$ may serve as a useful supplementary indicator set for preliminary reservoir-quality evaluation and favorable-interval screening. The proposed workflow therefore provides a pore-structure-based framework that may assist preliminary reservoir-quality assessment in tight carbonate gas reservoirs^99^.

## Data Availability

The datasets generated and/or analysed during the current study are not publicly available due to confidentiality restrictions associated with the funded research projects, but are available from the corresponding author on reasonable request.
